# Slide-free FIBI histology for improved quantitative vascular network analysis in breast cancer: Integrating vessel geometry and topology metrics

**DOI:** 10.1016/j.jpi.2026.100697

**Published:** 2026-07-22

**Authors:** Shael Brown, Nathan Anderson, Jazmin Orozco, Richard Levenson

**Affiliations:** a 84 Albert St., London NW1 7NR, UK; b UC Davis Health, Sacramento, CA 98517, USA

**Keywords:** Microscopy, Slide-free histology, Vasculature, Quantitative image analysis, Topological data analysis

## Abstract

Digital pathology increasingly seeks to extract quantitative vascular and microenvironmental features from routine histology images, but thin-section hematoxylin and eosin (H&E) slides can fragment vessels and obscure their spatial organization, constraining downstream computational analysis. Slide-free fluorescence-imitating brightfield imaging (FIBI) produces histology-like images directly from fresh or fixed tissue or from already prepared paraffin blocks within minutes and can better preserve apparent microvascular continuity in breast cancer and other specimens. In this exploratory digital pathology study, we acquired paired FIBI and H&E images from paraffin-embedded breast tissue blocks, manually segmented blood vessels in tumor and tumor-adjacent stroma, and quantified vascular architecture using both standard geometry-based metrics and topology-derived descriptors of inter-vessel arrangement, including a persistent-homology-based “vessel spacing” metric that captures multiscale clustering. Here, geometry refers to properties of individual vessels (size, length, and branching), whereas topology summarizes how vessels are arranged as a network, including how closely or loosely they cluster. FIBI images exhibited an easily appreciable increase in vascular information compared with matched H&E slides, with vessels appearing more continuous and more clearly resolved in both geometry (e.g., larger area, more branched, etc.) and network topology (e.g., decreased inter-vessel separation). Motivated by this qualitative impression of increased informational content, the goal of this study was to quantitatively assess and validate these differences by contrasting complementary geometry and topology-derived metrics of vessels in pairs of H&E and FIBI images. In particular, persistent homology-derived topology metrics provided information that was not linearly explained by standard geometric descriptors. Together, these findings demonstrate a practical digital pathology pipeline that integrates slide-free FIBI acquisition, vessel annotation, geometry- and topology-based feature extraction, and suggest that FIBI-derived vascular signatures, particularly when coupled with automated vessel segmentation, may provide informative input for future computational and artificial intelligence-based pathology models, and potentially clinical applications.

## Introduction

Clinical evaluation of tissue masses suspected of being cancerous increasingly combines radiological imaging^1^ with histopathological assessment, but definitive diagnosis, grading, and treatment decisions still rely on microscopic examination of tissue that has been paraffin-embedded, sectioned, stained with hematoxylin and eosin (H&E), and mounted on slides. Examination of H&E-stained slides under brightfield microscopy remains the clinical gold-standard for determining tumor type, grade, and prognosis and therapy options.^2^, ^3^ Traditionally, these decisions have relied on qualitative visual examination supplemented by molecular tests, but quantitative measures have become central as digital pathology and whole-slide imaging have matured. A familiar example is the prostate carcinoma, where both qualitative architectural patterns (Gleason grading)^4^, ^5^ and quantitative measurements such as mitotic count or nuclear size contribute to tumor grading. However, there is active interest in expanding the repertoire of robust, quantitative histological features, particularly in the context of computational and artificial intelligence (AI)-based approaches.

Vascular architecture is a key component of the tumor microenvironment. In breast cancer and other malignancies, microvessel density, vessel caliber, branching patterns, and spatial relationships between tumor and stroma have been linked to angiogenesis, growth, and metastatic potential.^6^, ^7^, ^8^, ^9^, ^10^ Prior work, particularly using angiography and radiology-based techniques, has shown that measures such as vessel tortuosity can help distinguish malignant from benign lesions and characterize tumor aggressiveness. Translating these concepts into routine histology remains challenging: thin sections can truncate vessels and reduce apparent continuity, and manual assessment of complex vascular networks is laborious and subjective.

Standard histology workflows also impose practical constraints. Preparing slides can take from hours to days or longer depending on lab resources, delaying availability of diagnostic or research-grade material. These limitations have motivated the development of slide-free imaging approaches that can rapidly generate diagnostic-quality histology images directly from fresh or fixed tissue, thereby providing earlier digital input for both human review and computational analysis.

Fluorescence-imitating brightfield imaging (FIBI) is one such method, generating H&E-like images from hand-cut thick tissue specimens within 3–5 min using relatively simple hardware.^3^, ^11^ FIBI employs 405-nm LED illumination in epifluorescence mode to excite subsurface autofluorescence, which backlights a tissue surface that had been quickly (∼1 min) stained with H&E.

**Fig. 1:**
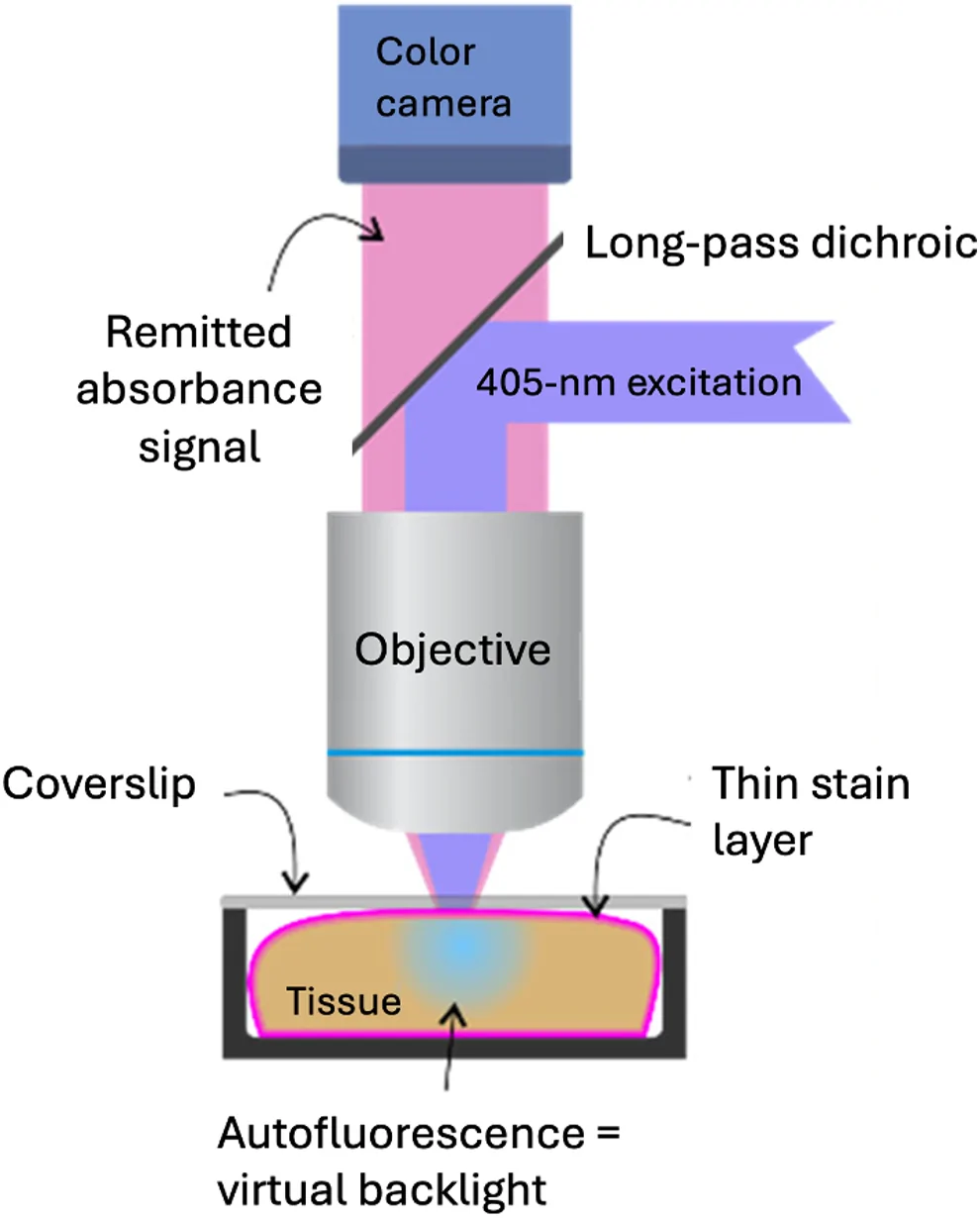
FIBI optical schematic. Components comprise a 405-nm LED excitation source, a standard microscope objective (typically 10× or 20×), a sample holder with a coverslip against which a tissue specimen is gently compressed for surface flattening, a 430-nm long-pass dichroic, and a CMOS RGB color camera. The tissue has been quickly (∼1 min) stained with hematoxylin and eosin before being inserted in the sample holder.

**Fig. 2:**
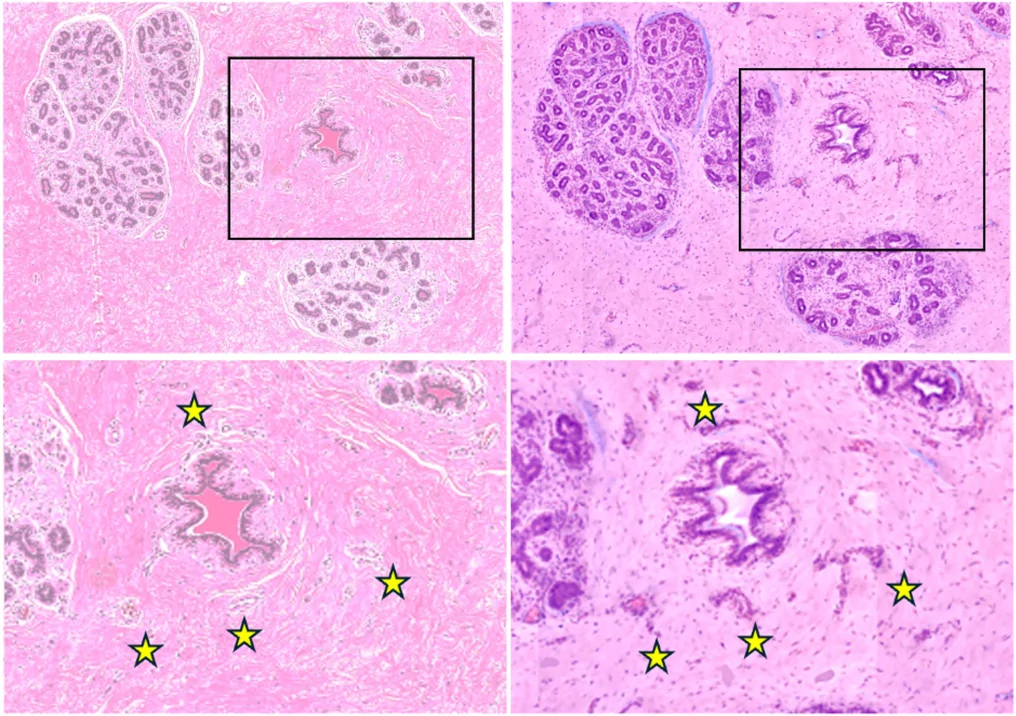
Examples of images of H&E-stained slides (left) and corresponding FIBI images taken from the surface of matched paraffin blocks of a specimen of benign breast. Whereas not completely identical, the image pairs resemble serial sections in terms of histology similarity. Close-ups of the boxed regions indicate that blood vessels are more easily appreciated in the FIBI image.

The resulting images resemble conventional H&E-stained slides as shown in Fig. 2. Because FIBI is employed on thick (not microtomed) tissue sections, certain structural features such as collagen bundles and (discussed here) blood vessels appear to be easier to evaluate in terms of their continuity, distribution, and other spatial features. (See Fig. 1.)

From a digital pathology perspective, FIBI offers two potential advantages. First, it can provide rapid, slide-free access to histology-like images suitable for standard whole-slide viewing and downstream computational pipelines. Second, by sampling a thicker tissue volume, FIBI may preserve microvascular networks in a way that improves quantification of vessel geometry and spatial organization compared to thin-section H&E. If true, this could yield richer feature sets for machine-learning models that incorporate vascular and microenvironmental information; accordingly, this study aims to identify and evaluate features, based on vessel geometry and topology, that may be informative for such applications.

Quantitative characterization of vasculature can be broadly divided into two complementary domains: geometry, which measures continuous features such as vessel length, area, and branching angles,^10^, ^12^ and topology, which captures the global spatial organization of vascular networks. Informally, geometry tells us what individual vessels look like, whereas topology asks about the “shape” of the entire vascular network—how vessels cluster, separate, or form gaps and loops when considered all together. Existing quantification approaches, especially in imaging and angiography, have emphasized geometric descriptors, such as microvessel density, vessel area, length, and tortuosity. Whereas such metrics capture properties of individual vessels, they do not fully describe the topology of how vessels are arranged relative to each other or how they form networks of branches and loops. Specifically, we focused on persistent homology (“PH”), a tool from the field of topological data analysis (“TDA”) that summarizes how connected components and loops appear and disappear across spatial scales in so-called persistence diagrams.^13^, ^14^ This approach has been applied successfully to tumor grading, microenvironment analysis, and other biomedical image domains.^15^, ^16^, ^17^, ^18^, ^19^, ^20^, ^21^, ^22^ A preliminary study provided encouragement that it might be useful.^23^

In the present study, we implement a digital pathology workflow that combines slide-free FIBI acquisition, whole-slide H&E scanning, manual vessel annotation in QuPath, and geometry- and topology-based feature extraction to characterize breast tumor vasculature. By imaging the paraffin-block face after sectioning for conventional slide preparation, we obtain paired FIBI and H&E views of near-identical regions. We then compare vascular metrics between modalities and assess whether FIBI-derived features, including topology-derived descriptors of inter-vessel organization, improve discrimination between tumor and tumor-adjacent stroma. Our aim is not to establish a prognostic model but to evaluate whether FIBI provides richer vascular information for computational pathology and whether topology-based metrics capture and quantify some of this added visual information, thereby adding value beyond standard morphometrics in this context.

## Method

This study was implemented as a digital pathology pipeline comprising: (i) acquisition of paired slide-free FIBI images and conventional H&E whole-slide images from the same paraffin blocks; (ii) manual zoning and vessel annotation in the open-source platform QuPath; (iii) export of annotation masks and construction of paired FIBI–H&E datasets at a matched pixel scale; (iv) extraction of vessel-level geometry metrics using Python-based libraries; and (v) computation of topology-derived features using PH, followed by mixed-effects and logistic regression models to assess modality differences and tissue-zone discrimination. All analysis code is made publicly available to facilitate reuse and extension in other digital pathology contexts.

Common abbreviations and analytic terms are summarized below (Table 1):

**Table 1:** Table of terms and abbreviations.

Term (abbr./alias)	Description
Hematoxylin & eosin-stained slides (H&E)	The conventional method of tissue preparation for histopathology.
Fluorescence imaging brightfield imaging (FIBI)	A slide-free microscopy method.
Topological data analysis (TDA)	Computational tools for quantifying the “shape” of a dataset.
Persistence diagram	A summary of shape features, components, and loops: Each feature represented by a pair of numbers (*birth*, *death*).
Persistent homology (PH)	An algorithm for computing persistence diagrams.
Vascular topology	The spatial arrangement of multiple vessels.

### Tissue sources and selection

Formalin-fixed, paraffin-embedded (FFPE) breast tissue blocks (*n* = 10) were obtained from the UC Davis Department of Pathology and Laboratory Medicine under IRB approval. Nine specimens originated from mastectomy or lumpectomy procedures diagnosed as invasive mammary carcinoma of various grades; four also contained ductal carcinoma in situ. One benign reduction-mammoplasty sample was included as a non-neoplastic example but was excluded from main analysis comparing FIBI vs. H&E, which focused on breast cancer specimens. Blocks were selected based on three histological criteria observable on archival slides: (1) histology consistent with the reported diagnosis; (2) adequate relevant tumor content; and (3) presence of sufficient dense tumor and neighboring stromal regions suitable for paired comparison.

### H&E histology and matched FIBI imaging

From each block, a new 4-μm section was cut and stained with H&E. Resulting slides were scanned in brightfield mode at 20× using a Leica/Aperio AT-2 scanner. FIBI imaging: After sectioning, the resulting block surfaces were treated with a gradient of solvent baths (xylene, 100% ethanol, 70% ethanol, and 35% ethanol) to dissolve paraffin wax (to a depth of a few hundred microns) before being rehydrated in 10% neutral buffered formalin. Each block surface was stained with hematoxylin (StatLab HXMMHLT) for 15 s, rinsed in deionized water, stained with eosin Y (StatLab STE0250) for 30 s, and rinsed again. Immediately after staining, block faces were imaged at 20× on a custom FIBI microscope.

### Digital image annotation

Blood vessels were manually annotated by trained researchers under pathologist supervision, following consistent criteria for inclusion (luminal vascular structures with endothelial lining) and exclusion (lymphatic channels, artifacts, and non-vascular spaces). These manual annotations serve as a reference standard for subsequent computational analysis and are intended as a foundation for future development of automated vessel-segmentation algorithms within similar digital pathology workflows.

**Fig. 3:**
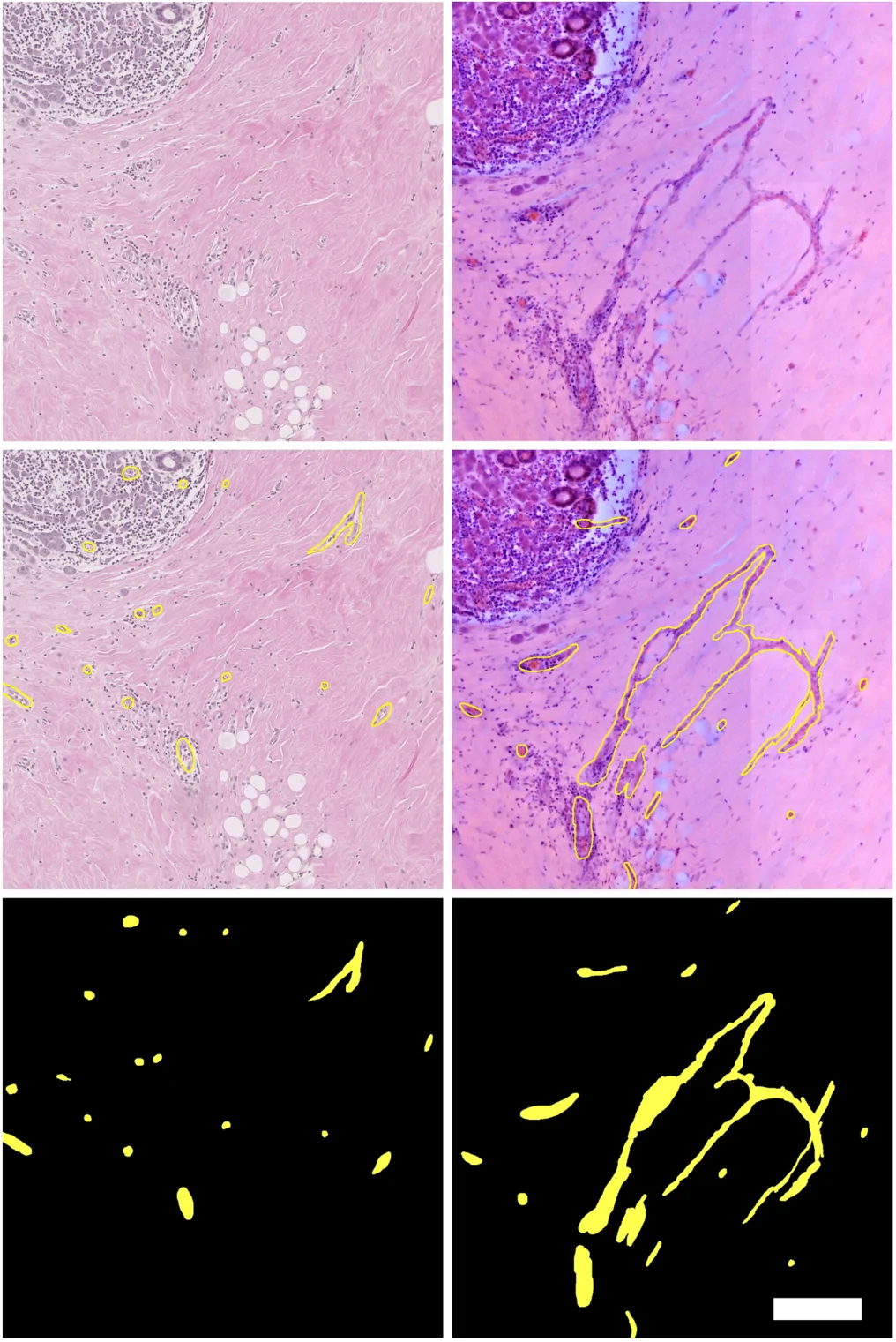
FIBI images can reveal more continuous and more branching vasculature than corresponding H&E images. Fig. 3A: a 4-μm-thick section, H&E-stained region adjacent to breast cancer; Fig. 3B: FIBI image of the surface of the matching paraffin block. A portion of a tumor deposit with inflammation is visible in the upper left corner. Fig. 3C and D highlight the vasculature that was manually segmented for downstream spatial data analysis. Finally, Fig. 3E and F show the raw annotation data on which analysis was performed. Scale bar = 200 μm.

FIBI and H&E images were imported into QuPath 0.6.0 for manual tissue zoning and vascular segmentation. Non-adjacent stroma and adipose deposits were excluded before highlighting regions of interest subdivided into *dense tumor* or *adjacent stroma* (not shown). Blood vessels were manually annotated by trained researchers under pathologist supervision. Annotation masks were exported as PNG files and downsampled to balance image scale and computational load. Finalized annotation images were matched to a scale of 1.0054 μm/pixel; this was achieved by downsampling the H&E whole-slide images by a factor of 2.0 and the FIBI images by a factor of 4.8. Paired, co-registered datasets were then used for quantitative analyses (see Fig. 3).

### Calculation of geometry and topology metrics

**Fig. 4:**
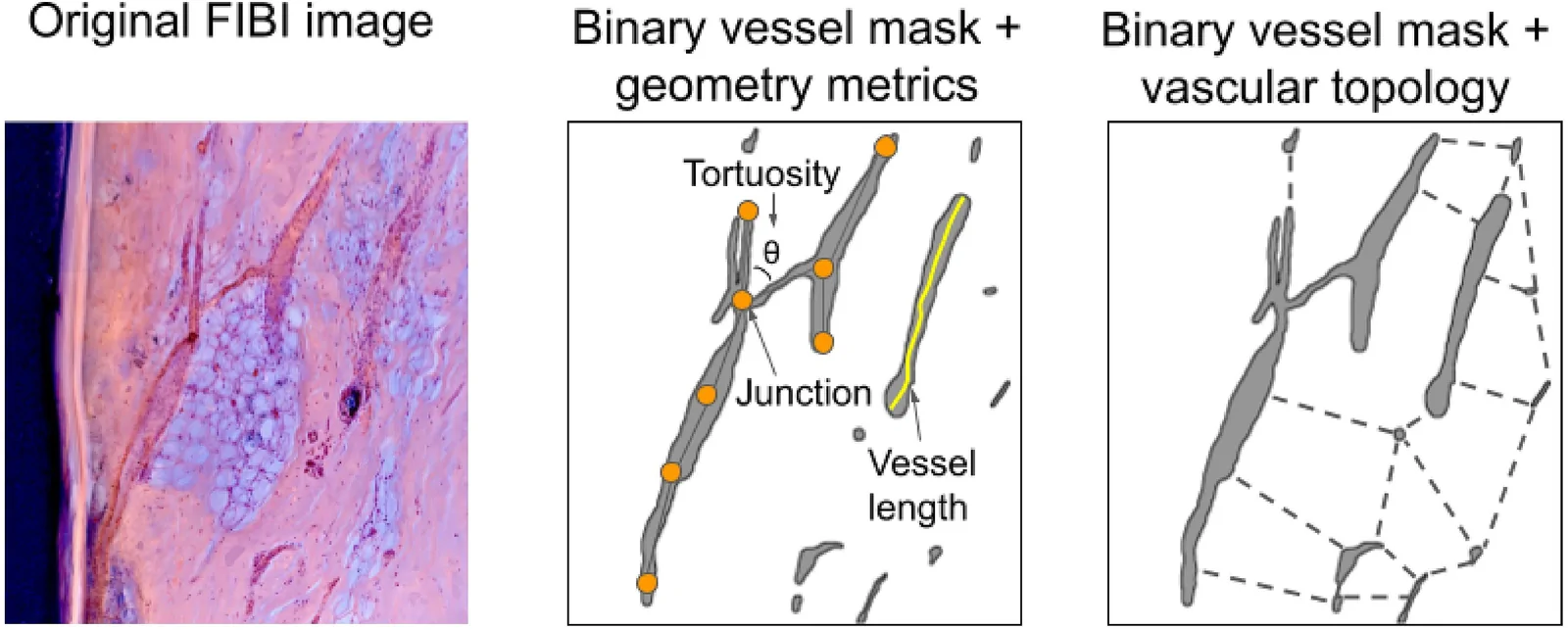
An original FIBI image and illustrations of some computed geometry metrics and vascular topology. Left: a portion of a FIBI image taken from a vessel-rich region. Center: three geometry metrics computed from binary vessel annotations. (i) A computed vessel graph is overlaid on top of one vessel with one junction and one branching angle labelled (nodes are junctions, segments along long straight portions or endpoints), and (ii) a vessel length is highlighted in yellow. Right: a subset of inter-vessel distances used for vascular topology metrics overlaid on top of the binary vessel annotation mask. (For interpretation of the references to color in this figure legend, the reader is referred to the web version of this article.)

Distinction between individual vessel geometry metrics and topology metrics (capturing vessel to vessel spatial arrangement) can be appreciated from Fig. 4.

### Geometry metrics

Individual vessel-level geometric properties (shown in Fig. 4) were computed in Python using scikit-image v0.21.0^27^ and skan v0.11.1^28^, as summarized in Table 2.

**Table 2:** Geometry metrics calculated from vessel annotations. The vessel annotation masks were first split into connected components (i.e., distinct vessels) and metrics were computed for each component.

Geometry metric	Description	Units
Microvessel density (MVD)	Number of vessels per unit area^13^	(μm)^−2^
Area	Average vessel area^14^	(μm)^2^
Perimeter	Average vessel perimeter^30^	μm
Circularity	Average ratio of (4π area/perimeter^2^) across vessels^30^	–
Length	Average vessel length, calculated as distance along a vessel centerline skeleton^14^	μm
Tortuosity	A measure of branching angle acuteness (i.e., extremeness) in vessel graphs based on the sum of angles metric (SOAM) averaged over all length-4 paths^12^, ^30^	–
Degree	The average number of branches per node in a vessel graph^14^, ^15^	–
Junctions	Average number branching points in a vessel graph^14^, ^15^	–

All metrics were averaged per specimen, by zone or over combined zone areas.

Vessel length was computed from a centerline representation of each binary vessel annotation using the scikit-image morphology.skeletonize function. Tortuosity, node degree, and junction counts were derived from graph representations of the vessel skeletons generated using the skan Skeleton function. These graphs iteratively prune non-branching pixels, resulting in a simplified network that preserves only branch points and endpoints. Tortuosity was quantified using local curvature along the skeleton. For each length-4 path of connected pixels, angular changes between successive segments were computed in both planar (in-plane) and torsional (out-of-plane) components. These were combined into a curvature measure for each central pixel, and overall tortuosity was calculated as the normalized sum of these curvature values across all such paths in the network. For further details on calculating tortuosity, see Bullitt et al.^8^

### Topology-derived metrics

From a pathology informatics standpoint, these persistence-based metrics provide multiscale, quantitative summaries of how vessels cluster and form loops, which can be used as engineered features in classification and prediction models alongside conventional morphometric descriptors. In this work, all reported topology effects derive from the H0 (connected-component) diagrams; H1 (loop-based) metrics were computed but did not show significant modality differences and are summarized in the Supplementary Materials.

PH computes shape features via a process called Vietoris Rips filtration.^24^ Vessels are connected at increasing distance thresholds to track the appearance (birth) and disappearance (death) of components (called *H*_0_ features) and loops (*H*_1_ features). To preserve biologically relevant branching information, inter-vessel distances were defined as the minimum Euclidean distance between pixels on each vessel annotation boundary, rather than centroid–centroid distances, a typical approach in TDA analyses of cell annotations.^18^, ^25^, ^26^ In the separate-zone analysis, we computed PH metrics for both distance approaches for comparison.

**Fig. 5:**
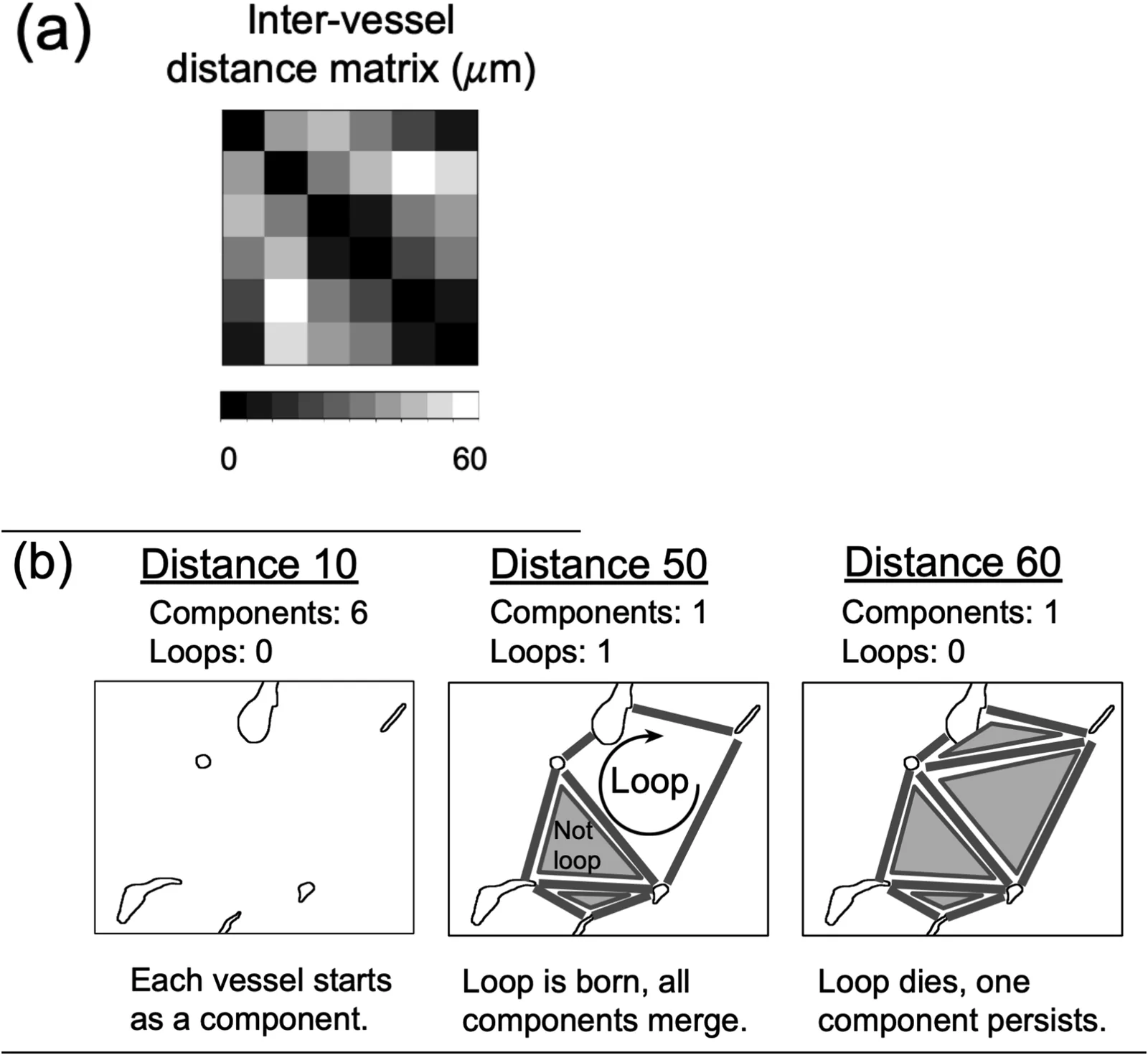
Computing persistent homology for vascular topology taken from one patch (seen in 5B) of a FIBI image. (a) The inter-vessel distance matrix, in arbitrary units, is the input to PH. Each row/column represents one of the six contiguous vessel annotations in the patch, and the matrix entry i,j is the smallest Euclidean distance between a pixel in vessel i and a pixel in vessel j. (b) The PH algorithm connects vessels at various increasing inter-vessel distances and tracks the appearance and disappearance, as a function of distance, of components and loops. At a small distance, for example, 10 units, each vessel constitutes a separate component (and therefore no loops are present). At a larger distance (50 units), all individual components have merged to create a single component, and a single loop (4-sided structure) is present. At a still larger distance (60 units), the loop has been filled in. The *H*_0_ persistence diagram for this example would consist of six pairs of (0, *death*) values, one of which would be ignored because the death value of one point is always infinity and therefore uninformative, and the *H*_1_ diagram would have a single pair (50, 60).

For each topological feature, PH records a *birth* value (the distance threshold when the feature first appears) and a larger *death* threshold (the distance threshold that fills in the hole that defines the feature), yielding a persistence diagram of 2D (*birth*, *death*) pairs for *H*_0_ components and *H*_1_ loops separately. In *H*_0_, the *birth* values are all 0 as each vessel begins the filtration process as its own component. The distribution of *death* values reflects the distance scales at which vessel components merge (i.e., components merge via edges between vessels), and the number of *H*_0_ features is one less than the number of vessels. In *H*_1_, *birth* values relate to the sampling density around each loop (a lower *birth* value indicates a higher sampling density) and *death* values approximate loop size; loops are filled in with triangles of connected vessels, which are, in the PH algorithm, not themselves loops. Persistence values, the *death* minus *birth* for each feature, is a measure of feature significance, with lower values suggesting that a feature represents topological noise. See Fig. 5 for an illustration of PH applied to a portion of a FIBI vascular topology.

The diagrams, *H*_0_ and *H*_1_, and their topology metrics (Table 3) were computed both for whole annotated images and within each separate tissue zone (tumor/adjacent stroma) using giotto-ph v0.2.1.^27^ For each zone, PH was computed on the full inter-vessel distance matrix rather than on smaller patches, so that topology metrics reflect global organization within that annotated region.

**Table 3:** Topology metrics calculated for vascular topologies.

Topology metric	Description	Units
Mean/max persistence	The mean/max persistence value computed from a persistence diagram.	μm
L2-norm (“vessel spacing”)	The square-root sum of squared persistence values in a persistence diagram.	μm

A large *H*_0_ maximum persistence indicates at least one vessel is unusually distant from all others, whereas a large mean persistence or L2-norm indicates vessels are more uniformly and widely separated. A large *H*_1_ maximum persistence indicates the existence of a significant loop structure, whereas a large mean persistence or L2-norm indicates multiple large loops.

A preliminary article explored the use of PH to compare vascular topology in a single pair of FIBI and H&E images: the results motivated the present, more detailed examination.^23^

### Statistical modeling framework

For both analyses, mixed effects regression models were fit with random effects for tissue samples to account for inter-subject biological variability. In the combined-zone analysis, each of the 14 metrics computed (raw and log-transformed) was linearly modeled, with imaging modality (FIBI vs. H&E) as the principal covariate. Bonferroni correction of the FIBI variable coefficient across all 14 metrics was applied to identify significant effects (correcting for false-discovery rate^28^ at the level 0.05 yielded nearly identical results). To determine whether significant topology metrics provided independent information, we subsequently fit a linear model predicting topology from geometry and assessed it for significance.

The separate-zone analysis was designed to determine whether the significant FIBI contrasts identified in the combined-zone topology improved distinction among tissue zones in terms of vascular phenotype. Each model in this stage was a logistic regression predicting zone label, included a random effect for tissue sample, and fixed effects for each vessel metric standardized by zone area. We first fit a model containing the geometry metrics, vessel spacing, the imaging modality variable (i.e., a variable that is 1 for zones from FIBI images and 0 for zones from H&E images), and the interaction terms between modality and each metric to identify significant interactions. We then simulated “removing” the FIBI contrasts by constructing a modified dataset in which, before zone area standardization, the metric values for FIBI zones were adjusted by reversing (i.e., dividing by) the estimated percent difference effects of each significant contrast. Using this adjusted dataset, we fitted a new logistic regression model and compared its Akaike Information Criterion (AIC value) with that of a baseline model fit to the original data.

## Results

### Overview of dataset and analyses

Paired FIBI–H&E images subdivided into *dense tumor* and *tumor-adjacent stroma* zones were analyzed, and individual blood vessels, including capillaries and larger structures, were manually segmented. The resulting annotation masks were subjected to quantitative analysis using geometry- and topology-based metrics to examine the hypothesis that FIBI images reveal more complete vascular information than matched conventional H&E slides.

Two complementary analyses were performed. A combined-zone analysis assessed all regions together to quantify direct modality differences, and separate-zone analysis evaluated how zone metrics related to the zone label of tumor vs. adjacent stroma. This dataset and analysis pipeline illustrate a practical use case for combining slide-free imaging with feature engineering in a digital pathology environment.

### Visual and quantitative comparison

**Fig. 6:**
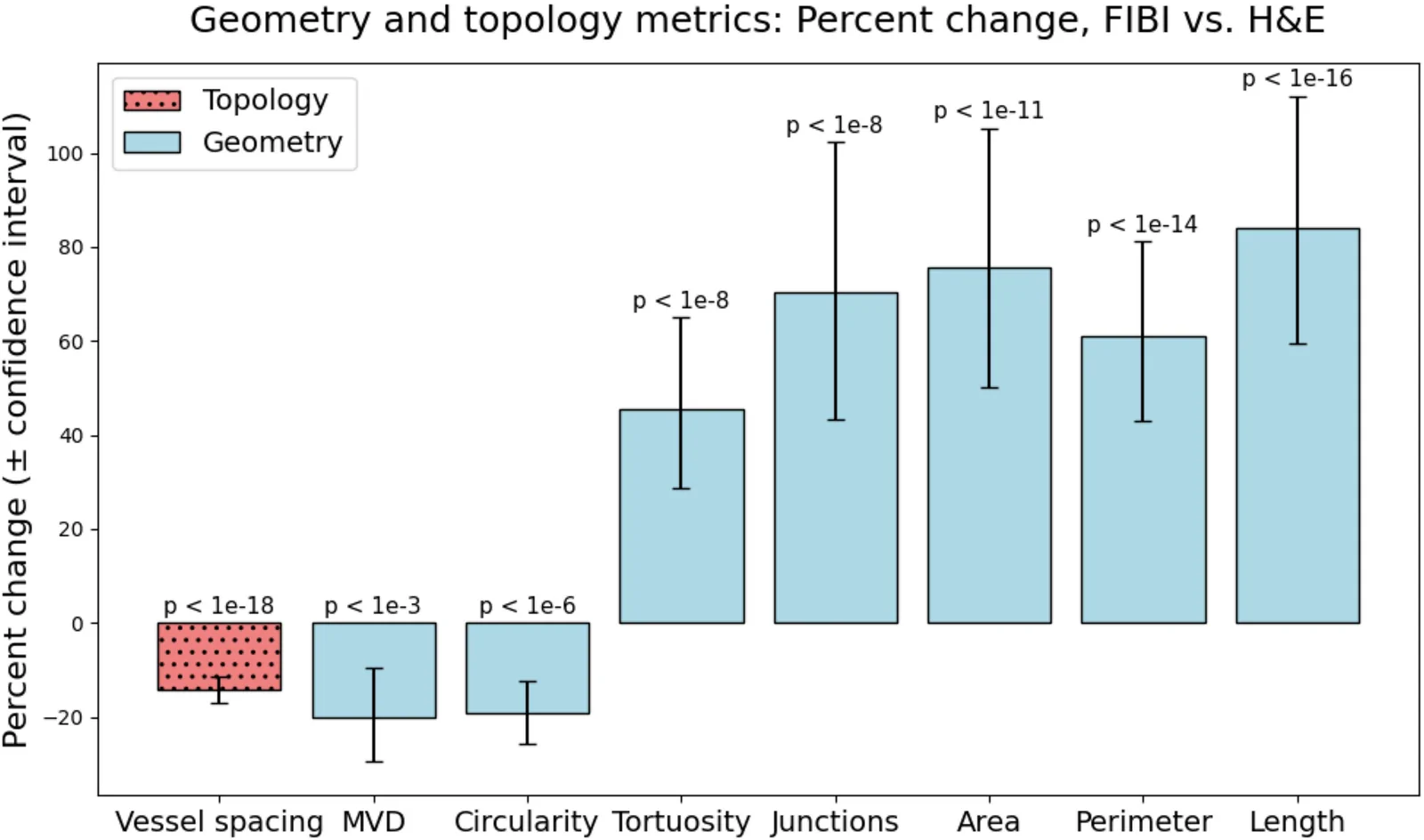
Comparison of topology (red) and geometry (blue) metrics between H&E and FIBI images. Each bar represents the exponentiated coefficient from the log-scale model with 95% confidence intervals, and *p*-values are shown above the error bars; the topology metric is characterized by the smallest *p*-value. Metrics such as the mean/max persistence metrics and L2-norm of H_1_ that did not exhibit significant differences between H&E and FIBI were excluded from the plot. (For interpretation of the references to color in this figure legend, the reader is referred to the web version of this article.)

Visual inspection of matched H&E–FIBI pairs reveals that FIBI images consistently exhibit more continuous and branched vasculature. Linear modeling confirmed these qualitative impressions. Among 14 evaluated metrics, FIBI showed significantly greater *vessel area*, *perimeter*, *tortuosity*, *length*, and *number of junctions*, and significantly lower *MVD, circularity*, and *vessel spacing* (Fig. 6), due to the increased subsurface detail visible with the non-thin–sectioned FIBI specimens. Vessel degree (branches per node) was also significantly greater in FIBI images, but only by about 5%, which led to its exclusion from the plot. Percent changes were modeled to plot and compare variables of differing units and scales; for raw-scale effects see *Supplementary Data Table 1*.

Vessels were closer (14% lower *vessel spacing*); fewer (20% lower *MVD*); more complex (20% less *circular*; 46% more *tortuous* and 70% more *junctions*); and larger (75% more *area*, 61% more *perimeter* and 84% *longer*). The “vessel spacing” topology metric showed the smallest absolute effect but also displayed the smallest *p*-value, implying robust reliability compared with geometric metrics. The raw-scale differences (Supplementary Data Table 1) only differed from the results presented here in that the MVD was no longer significant at the Bonferroni level, and Circularity did not admit a *p*-value as its model failed to converge.

To assess the stability of the metric effects given the limited sample size, we repeated the modeling procedure across 1000 bootstrap resamples. The resulting bootstrap 95% confidence intervals closely matched the original intervals, particularly for metrics with significant differences between FIBI and H&E, supporting the robustness of these findings and suggesting they may generalize to unseen images.

The linear models, one for each metric, determined significant modality differences, but could not rule out linear dependencies between geometric and topological differences. To assess whether topology effects were merely linear functions of geometry, we fit a linear model predicting vessel spacing from the geometry metrics and found no significant coefficients, no overall significant linear effect (*p*-value of the model F-statistic), and an adjusted $R^{2}$ near zero, supporting the view that vessel spacing captures information not explained by the geometric descriptors deployed here.

### Relating FIBI variables to tissue zones

Although inter-modality comparisons quantify differences in vascular architecture, it is not immediately clear whether these differences correspond to biologically meaningful variation. In the interaction model, all metric coefficients were highly significant except for area and the FIBI–MVD interaction. When the metrics were left unstandardized, however, all significant effects disappeared, underscoring the importance of zone area as a scaling factor. The significant interaction terms in the standardized model indicate that the metric differences observed in FIBI zones could be providing meaningful predictive information. Finally, comparison of AIC values between the baseline model and the model fit to the FIBI-effect–removed dataset showed a difference of 3.7 in favor of the baseline model, consistent with a modest loss of information when modality-specific metric contrasts are removed from FIBI images.^29^ In other words, the additional vascular information captured in FIBI images contributes measurable discriminatory signals for computational separation of tumor from tumor-adjacent stroma. Although separating tumor from adjacent stroma is itself a relatively straightforward task, these results suggest potential value of FIBI-derived vascular metrics as input features for downstream predictive models in more biologically complex settings.

### Summary of key findings

Compared with H&E sections, FIBI-visualized vasculature was characterized by fewer, larger, and more complex vessel structures that were located closer together. These results indicate that FIBI can better capture vascular histology signals compared to conventional H&E, and that TDA provides complementary measurements of this added information.

## Discussion and conclusions

FIBI can generate histology-like images within minutes of tissue acquisition, and prior work has shown that its appearance is sufficiently similar to conventional H&E to support routine diagnostic interpretation. In the digital pathology setting, however, a key question is not only whether FIBI can replace slides visually but whether it also provides richer or more informative input data for computational analysis. In this study, we implemented a practical pipeline combining slide-free FIBI acquisition, whole-slide H&E scanning, manual vessel annotation, and geometry- and topology-based feature extraction to quantify breast tumor vasculature. The quantitative results confirm and extend qualitative impressions: compared with matched H&E sections, FIBI consistently captured larger, more continuous, and more complex vascular patterns, particularly evident in Fig. 3, that translated into distinct geometric and topology-derived metrics, whereas still producing essentially 2D projections of a surface layer rather than true volumetric reconstructions. Whereas the present study relied on paraffin-block-face imaging to maximize the similarity of fields of view between the paired FIBI and H&E images, in the future, FIBI would be employed on tissue specimens, fresh or fixed, that have not undergone paraffin embedding, thus providing near-real–time diagnostic results.

It is important to emphasize that the observed differences between FIBI and thin-section H&E likely arise from multiple sources. Thick-surface imaging may preserve apparent continuity of individual vessels that are truncated in 4-μm sections, but it may also merge closely apposed small vessels, alter contrast because of surface staining differences, and introduce projection effects, where structures at slightly different depths are superimposed. In addition, the FIBI and H&E images represent closely matched but not perfectly co-registered tissue planes, so some differences in topology may reflect section choice rather than modality alone; however, the consistent direction and magnitude of effects across specimens argue that modality-specific sampling plays a substantial role. Additionally, we expect that the effects of small, random co-registration misalignments across samples would average out in (generalized) linear models. Our results therefore cannot uniquely distinguish improved preservation of “true” vascular continuity from under-detection or merging of small vessels in FIBI, and we do not regard FIBI images as ground-truth network representations. Resolving these contributions will require volumetric comparisons with cleared-tissue three-dimensional (3D) or confocal imaging and direct correlation with vascular immunostains.

Much previous work identifying the biological significance potential of vascular structures relied on macroscopic tissue imaging in 3D, typically accomplished by various radiology-based techniques including angioscopy. However, high-quality 3D vessel capture can also be achieved via advanced microscopy approaches, such as light-sheet microscopy (LiSM) and confocal microscopy.^30^, ^31^ In this context, FIBI occupies a distinctive niche. Whereas LiSM and confocal offer volumetric acquisition and fluorescence-based quantification (mean fluorescence intensity, surface curvature, and related descriptors), FIBI achieves high-fidelity histological imaging with single-image output, minimal sample preparation, substantially lower equipment cost, and near-real–time acquisition. However achieved, the vessel imaging provided by FIBI reveals complex structures that require appropriate tools for analysis. We applied both geometry- and topology-based analytics and demonstrated that these approaches appear to be complementary. Practically, this means that in addition to measuring what each vessel looks like, we also quantify how vessels are arranged as a group, i.e., whether they are tightly packed or more widely separated; in this way, global vascular organization metrics can complement conventional morphometrics in downstream models.

Among the *topological* features examined, only vessel spacing, a measure reflecting degree of spatial clustering (derived from the H₀ persistence diagram), differed significantly between imaging modalities, quantifying certain visual differences in global vasculature. In contrast, metrics capturing loop-like structures (H₁ persistence) did not show significant differences. This likely reflects two factors. First, apparent gaps in true loops may arise largely from sectioning of inherently 3D vasculature. Second, although vessels in FIBI tend to appear larger and more continuous, these changes may shift both the formation and closure of loops, computed from images to smaller spatial scales, resulting in little net change in persistence-based loop metrics. These results cannot distinguish between improved preservation of vessel continuity and potential under-detection or merging of small adjacent vessels in FIBI; future studies with volumetric ground truth and vascular markers will be needed to resolve this.

This exploratory work has several limitations that define clear directions for further study. First, the cohort is small and restricted to breast tissue from a single institution, and the behavior of FIBI-derived vascular metrics in other organs and staining protocols, particularly in densely structured tissues such as liver or kidney, remains unknown. Second, vessel annotations were generated manually by trained researchers under pathologist supervision, which is feasible for method development but not for routine deployment; robust, reproducible automated vessel segmentation and quantitative assessment of inter-observer and inter-algorithm agreement will be essential for scaling this workflow. Third, we analyze 2D projections of a thick tissue surface layer, rather than true volumetric datasets, and cannot yet separate improved preservation of apparent vessel continuity from projection and staining effects without 3D ground truth. Finally, the geometry and topology signatures reported here have not been linked to specific diagnostic or prognostic tasks, such as detection of lymphovascular invasion or outcome prediction. Larger, outcome-annotated cohorts and task-focused studies will be required to determine the practical utility of FIBI-based vascular features in computational pathology; studies focusing on breast and prostate cancer are being planned at UC Davis.

From a pathology informatics perspective, these findings have two main implications. First, they suggest that slide-free FIBI images can provide a higher-quality substrate for vascular feature extraction than thin-section H&E alone, by preserving vessel continuity and network structure. Second, they demonstrate that combining standard geometrical descriptors with topology-derived metrics, effectively combining what each vessel looks like with how vessels are arranged as a network, yields a richer feature space that can enhance computational separation of tissue zones and could be incorporated into future machine-learning models for grading, risk stratification, or response prediction. The open-source code and analysis framework presented here provide a template for extending similar workflows to other tissue types, imaging modalities, and quantitative endpoints. However, caution is indicated: Current applications of TDA to histopathology can be limited by sensitivity to preprocessing and patching strategies, single-institution cohorts, dependence on segmentation quality, and challenges in anatomically interpreting persistence-based features, underscoring the need for standardized pipelines and external validation before routine deployment.^32^

If FIBI continues to show enhanced representation of biologically significant histological features at a fraction of the cost and time of conventional H&E, it could play a useful role as a rapid digital input for computational pathology pipelines. Researchers would gain access to more continuous, information-rich vascular and microenvironmental features for algorithm development, whereas clinical workflows could benefit from faster, cost-effective tissue evaluation without sacrificing diagnostic confidence. In combination with automated segmentation and advanced modeling, the integration of FIBI imaging and quantitative feature engineering, including topology-based descriptors, offers a promising route toward more informative and scalable digital pathology.

## Declaration of competing interest

The authors declare the following financial interests/personal relationships which may be considered as potential competing interests:

Richard Levenson reports financial support was provided by the National Institutes of Health: R33CA278544--Joint IMAT-ITCR Supplement. Richard Levenson reports a relationship with MUSE Microscopy Inc. that includes: equity or stocks. Richard Levenson has patent Fluorescence Imitating Brightfield Imaging (FIBI) issued to UC Davis. The other authors declare that they have no known competing financial interests or personal relationships that could have appeared to influence the work reported in this article.

## Appendix A Supplementary data

Supplementary material: Geometry and topology metrics

## References

[1] Frangioni J.V. (2008). New technologies for human cancer imaging. J Clin Oncol Off J Am Soc Clin Oncol.

[2] Tseng L.J., Matsuyama A., MacDonald-Dickinson V. (2023). Histology: the gold standard for diagnosis?. Can Vet J Rev Vet Can.

[3] Au Yeung S., Giaretta P., Morningstar T. (2023). Utility of fluorescence imitating brightfield imaging microscopy for the diagnosis of feline chronic enteropathy. Vet Pathol.

[4] Chen N., Zhou Q. (2016). The evolving Gleason grading system. Chin J Cancer Res Chung-Kuo Yen Cheng Yen Chiu.

[5] Rani E., Nibhoria S., Nagpal N. (2023). Outlook of Gleason score in prostate carcinoma and correlation with PSA levels: a study in a tertiary care hospital. J Cancer Res Ther.

[6] Liu L., Zhang Y., Liu H. (2025). Transforming cancer therapy: unlocking the potential of targeting vascular and stromal cells in the tumor microenvironment. Cancer Res.

[7] Dvorak H.F. (2015). Tumor stroma, tumor blood vessels, and antiangiogenesis therapy. Cancer J.

[8] Bullitt E., Gerig G., Pizer S.M., Lin W., Aylward S.R. (2003). Measuring tortuosity of the intracerebral vasculature from MRA images. IEEE Trans Med Imaging.

[9] Weidner N. (1995). Intratumor microvessel density as a prognostic factor in cancer. Am J Pathol.

[10] Dolezyczek H., Rapolu M., Niedzwiedziuk P. (2020). Longitudinal in-vivo OCM imaging of glioblastoma development in the mouse brain. Biomed Opt Express.

[11] Borowsky A.D., Levenson R.M., Gown A.M. (2024). A pilot validation study comparing fluorescence-imitating brightfield imaging, a slide-free imaging method, with standard formalin-fixed, paraffin-embedded hematoxylin-eosin–stained tissue section histology for primary surgical pathology diagnosis. Arch Pathol Lab Med.

[12] Plummer C.F. (2024). Identifying Graph Characteristics in Growing Vascular Networks. http://urn.kb.se/resolve?urn=urn:nbn:se:uu:diva-524588.

[13] Hatcher A. (2000).

[14] Edelsbrunner, Letscher, Zomorodian (2002). Topological persistence and simplification. Discrete Comput Geom.

[15] Yang J., Fang H., Dhesi J. (2025). Topological classification of tumour-immune interactions and dynamics. J Math Biol.

[16] Chulián S., Stolz B.J., Martínez-Rubio Á. (2023). The shape of cancer relapse: topological data analysis predicts recurrence in paediatric acute lymphoblastic leukaemia. PLoS Comput Biol.

[17] Vandaele R., Mukherjee P., Selby H.M., Shah R.P., Gevaert O. (2023). Topological data analysis of thoracic radiographic images shows improved radiomics-based lung tumor histology prediction. Patterns.

[18] Singh N., Couture H.D., Marron J.S., Perou C., Niethammer M., Wu G., Zhang D., Zhou L. (2014). Machine Learning in Medical Imaging.

[19] Aukerman A., Carrière M., Chen C., Gardner K., Rabadán R., Vanguri R. (2021). Persistent homology based characterization of the breast cancer immune microenvironment: a feasibility study. J Comput Geom.

[20] Lawson P., Sholl A.B., Brown J.Q., Fasy B.T., Wenk C. (2019). Persistent homology for the quantitative evaluation of architectural features in prostate cancer histology. Sci Rep.

[21] Bukkuri A., Andor N., Darcy I.K. (2021). Applications of topological data analysis in oncology. Front Artif Intell.

[22] Yan C., Nakane K., Wang X. (2020). Automated Gleason grading on prostate biopsy slides by statistical representations of homology profile. Comput Methods Prog Biomed.

[23] Xu M., Anderson N., Levenson R.M., Prasanna P., Chen C. (2025). Topol- Graph-Inf Imaging Inform First Int Workshop TGI3 2024 Held Conjunction MICCAI 2024 Marrakesh Moroc Oct 10 2024 Proc Int Workshop Topol- Graph-Inf.

[24] Zomorodian A., Carlsson G. (2005). Computing persistent homology. Discrete Comput Geom.

[25] Bhaskar D., Zhang W.Y., Volkening A., Sandstede B., Wong I.Y. (2023). Topological data analysis of spatial patterning in heterogeneous cell populations: clustering and sorting with varying cell-cell adhesion. NPJ Syst Biol Appl.

[26] Vipond O., Bull J.A., Macklin P.S. (2021). Multiparameter persistent homology landscapes identify immune cell spatial patterns in tumors. Proc Natl Acad Sci U S A.

[27] Pérez J.B., Hauke S., Lupo U., Caorsi M., Dassatti A. (2021). giotto-ph: a Python library for high-performance computation of persistent homology of Vietoris-Rips filtrations. arXiv.

[28] Benjamini Y., Hochberg Y. (1995). Controlling the false discovery rate: a practical and powerful approach to multiple testing. J R Stat Soc Ser B Stat Methodol.

[29] Burnham K.P., Anderson D.R. (2004). Model Selection and Multimodel Inference.

[30] Lauwers F., Cassot F., Lauwers-Cances V., Puwanarajah P., Duvernoy H. (2008). Morphometry of the human cerebral cortex microcirculation: general characteristics and space-related profiles. NeuroImage.

[31] Oren R., Fellus-Alyagor L., Addadi Y. (2018). Whole organ blood and lymphatic vessels imaging (WOBLI). Sci Rep.

[32] Skaf Y., Laubenbacher R. (2022). Topological data analysis in biomedicine: a review. J Biomed Inform.

