# Supplementary material for: Slide-free FIBI histology for improved quantitative vascular network analysis in breast cancer: Integrating vessel geometry and topology metrics
Source: J Pathol Inform. 2026 Jul 22;22:100697. doi: 10.1016/j.jpi.2026.100697 (PMC13507840; doi:10.1016/j.jpi.2026.100697)
Supplement: Supplementary file 1 [file mmc1.docx]

**Supplementary Data**

| Metric | H&E value | FIBI value | Difference | Significance |
| --- | --- | --- | --- | --- |
| Vessels per unit area (MVD, vessels per mm^2^) | 7 | 6 | -1 | NS |
| Area (𝜇m^2^) | 1200 | 2100 | +900 | <0.001 |
| Perimeter (𝜇m) | 130 | 210 | +80 | <0.001 |
| Circularity | 0.66 | 0.53 | -0.13 | Not applicable |
| Tortuosity | 0.011 | 0.015 | +0.004 | <0.001 |
| Length (𝜇m) | 47 | 87 | +40 | <0.001 |
| Degree | 1.09 | 1.15 | +0.06 | <0.001 |
| Junctions | 0.25 | 0.40 | +0.15 | <0.001 |
| Mean persistence *H*_0_ | 126 | 122 | -4 | NS |
| Mean persistence *H*_1_ | 90 | 92 | +2 | NS |
| Max persistence *H*_0_ | 1060 | 980 | -80 | NS |
| Max persistence *H*_1_ | 990 | 820 | -170 | NS |
| L2 norm *H*_0_ (farness) | 3500 | 3000 | -500 | <0.001 |
| L2 norm *H*_1_ | 1440 | 1280 | -160 | NS |

Supplementary Table 1: Raw-scale differences of geometry (light blue) and topology (light red) metrics in FIBI images compared to H&E images, determined from linear models with random effects for tissue sample. Metrics without a significant difference (i.e., the model’s FIBI coefficient), at the Bonferroni correction level of 0.05/14 are labelled as “NS”. The H&E value is equivalent to the model intercept, and the FIBI value is the intercept plus the FIBI coefficient. Circularity did not admit a p-value because the model did not converge.
